# Susceptibility of Assessment Types to AI-Generated Content in Digital Health and Health Information Management Education: Quasi-Experimental Pilot Study

**DOI:** 10.2196/82988

**Published:** 2026-03-30

**Authors:** Tafheem Ahmad Wani, Michael Liem, Natasha Prasad, Kerin Robinson, Abbey Nexhip, Melanie Tassos, Stephanie Gjorgioski, Urooj Raza Khan, James Boyd, Merilyn Riley

**Affiliations:** 1 School of Psychology and Public Health La Trobe University Melbourne, Victoria Australia

**Keywords:** academic integrity, assessment design, ChatGPT performance, digital health education, generative artificial intelligence, health information management, quasi-experimental study

## Abstract

**Background:**

Generative artificial intelligence (AI) tools, such as ChatGPT, are increasingly used in higher education and have raised significant concerns about assessment validity and academic integrity. In Digital Health and Health Information Management (DIGHIM) programs, assessments are designed to evaluate a mix of technical skills, contextual reasoning, and professional judgment that underpin medical and health practice. Understanding how generative AI performs across different assessment types is, therefore, critical to identifying which formats are most susceptible to AI-generated content and how assessments may be redesigned to remain authentic and educationally meaningful.

**Objective:**

This study aimed to evaluate ChatGPT’s performance across diverse assessment types in DIGHIM education by examining how task complexity influences AI-generated output quality, and develop recommendations for ethical and effective AI integration in assessments.

**Methods:**

A pilot quasi-experimental design compared ChatGPT-generated responses with deidentified student submissions across 5 assessment types: digital health solution design, business case analysis, reflective assessment, SQL health database programming, and a health classification quiz. For each task, multiple AI submissions were produced using different prompting strategies, including rubric integration and the use of ChatGPT (GPT-4 and o1 Preview model). Blinded academic markers evaluated all AI-generated submissions and previously submitted deidentified student assessments against standard rubrics, and descriptive statistics were used to compare performance.

**Results:**

ChatGPT’s performance varied considerably across assessment types. It achieved its highest accuracy scores in objective, rule-based tasks such as multiple-choice quiz items in health classification (mean 88%, SD 0%) and produced well-structured, coherent responses for reflective assessments (mean 69%, SD 12.8%), though these often lacked personalization and nuanced industry context. In descriptive analytical tasks, such as digital health business cases and solution designs, ChatGPT produced logically structured work with reasonable use of evidence but failed to provide deep contextualization, domain-specific insights, or visual elements expected in DIGHIM practice. Technical assessments revealed the greatest limitations: SQL programming tasks averaged 42% (SD 17.2%) with persistent schema errors, incomplete queries, and weak interpretation of health data outputs, while scenario-based clinical coding scored just 7% (SD 0%), reflecting a lack of precision in applying *ICD-10-AM* (*International Classification of Diseases, Tenth Revision, Australian Modification*) rules and coding conventions. Structured prompting and rubric integration improved results, particularly in descriptive and reflective tasks (up to 80%), but the advanced o1 Preview model did not consistently outperform earlier versions.

**Conclusions:**

While ChatGPT performs well in structured, rule-based, and reflective tasks, it remains limited in technical accuracy, contextual reasoning, and applied DIGHIM competencies. To support academic integrity and workforce readiness, assessment design should prioritize critical thinking, ethical reasoning, and scenario-based problem-solving aligned with health care practice. Using AI as a tool for critique and refinement, rather than a substitute for student work, may help educators prepare learners for responsible AI use in medical and health professional education.

## Introduction

### Background

Artificial intelligence (AI) is a transformative technology that enables systems to generate outputs, such as content, recommendations, or decisions, based on human-defined objectives [1,2]. A key branch, generative AI (GenAI), produces human-like media (text, images, videos, and code) in response to prompts [3]. At the forefront are large language models (LLMs) such as ChatGPT (OpenAI), Microsoft Copilot, and Gemini (Google), which use deep learning and vast datasets to understand context, generate coherent responses, and adapt tone and style [4-8]. These models improve continuously through reinforcement learning with human feedback [9-11].

In higher education, LLMs have rapidly gained attention among academics and students [8,12-14], supporting assessment design, automated marking, and curriculum development [14-17], improving feedback timeliness and reducing grading errors [14,18,19]. They also help students refine language, generate ideas, and start research inquiries [20]. Overall, recent evidence in medical education highlights the rapid diversification of GenAI applications across learning resources, instructional methods, and assessment practices, offering tangible opportunities in areas such as documentation support, simulation-based training, and personalized learning [21-23].

Despite the benefits, the integration of GenAI technologies into higher education can present significant ethical challenges [24]. These include ChatGPT’s use of inaccurate content (including fictitious reference material) [25], concerns regarding human teacher replacement [11], and negative repercussions on students’ critical thinking and problem-solving skills [26]. Most significantly, the use of ChatGPT can pose a threat to academic integrity and ethics [27]. A recent 2024 scoping review has highlighted that traditional assessment methods do not operate effectively in GenAI-facilitated learning environments, prompting the need for innovative and refocused assessment designs that foster career-driven competencies and lifelong learning skills [28]. The ethical framework surrounding students’ use of AI in educational assessments primarily involves transparency, accountability, and reliability [29]. Transparency requires that students acknowledge the use of AI appropriately in their submissions. Students must also be accountable for the content they submit, as “outputs of AI tools can include biased, inaccurate, or incorrect content that users should be aware of” [29]. In some instances, GenAI can fabricate information, which raises the question of the reliability of the outputs [7].

The accessibility of AI tools and the difficulty in detecting AI-generated content [7,16] may tempt students to engage in academic misconduct. This raises concerns about assessment fairness, equity, and the credibility of academic credentials earned through digital platforms. Educators, therefore, face challenges in maintaining the integrity of assessments and ensuring educational quality [16,30]. Recent studies also report learners’ desire for clear institutional guidance on appropriate AI use, aligning with calls for explicit policy and training [31,32].

La Trobe University, Australia, offers a wide range of health programs. The Digital Health and Health Information Management (DIGHIM) programs provide formal education in disciplines that support contemporary health care and medical practice, aiming to develop students’ technical, professional, and interpersonal skills [33,34]. Typical enrolments include undergraduate and master’s-level students from clinical or quasi-clinical backgrounds such as nursing, allied health, health sciences, health administration, biomedical sciences, and related fields, as well as graduates seeking roles at the intersection of health care delivery, health data, and digital systems. The curriculum is aligned with medical education priorities, including clinical documentation, health classification, health data governance, digital health (DH) system design, and the ethical use of health information, all of which directly support contemporary medical practice and health service delivery. The DIGHIM programs emphasize discipline-specific and generic competencies, which are inclusive of problem-solving, critical thinking, and ethical decision-making that are typically assessed through academic essays, reports, multiple-choice quizzes, presentations, case studies, programming exercises, and practical simulations [35,36]. The proliferation of AI-generated content threatens the validity and reliability of assessments in most of these areas, potentially compromising the effectiveness of evaluating students’ comprehension and competence [16]. In this context, the broader medical-education literature now differentiates by task type and competency domain: from automated short-answer scoring and case generation to simulation and reflective work, strengthening the rationale for DIGHIM-specific analysis of assessment susceptibility and redesign [21,37-39].

Previous research on the impact of GenAI tools in higher education has primarily focused on specific types of assessments, such as multiple-choice questions or essay evaluations, mainly within the medical [40-42] and business fields [4]. For example, Chaudhry et al [16] undertook an evaluation of AI-generated assessments in a Bachelor of Business Administration, including case analysis, empirical study report, self-reflection, group work, and calculation-based assessments. These findings do not necessarily translate well to the more specialized fields of DH and health information management (HIM), each of which requires a distinct combination of technical, analytical, and decision-making skills. DIGHIM programs, for instance, demand expertise in areas such as health classification, epidemiology and biostatistics, DH solution design, health data governance, and interoperability [33,34]. These specialist areas of study necessitate a rigorous and diverse set of assessment methods, incorporating scenario-based problem-solving, data interpretation, and system implementation tasks that test both theoretical knowledge and practical application. Given the interdisciplinary nature of DH, which aims to bridge clinical practice, data science, and IT, there is a need for a separate evaluation of how GenAI interacts with these unique assessments [43]. This study responds to that gap by examining assessment type susceptibility in DIGHIM and aligning implications with contemporary redesign strategies and governance recommendations emerging from the 2024-2025 literature [21,31,32,44].

### Aims

This study examined how GenAI, specifically ChatGPT, performed across different DIGHIM assessment types and how its outputs compared with student work, with the aim of identifying assessment formats most susceptible to AI-generated content. It further sought to inform assessment redesign by identifying approaches that promoted authenticity, higher-order reasoning, and responsible AI use, thereby strengthening academic integrity through design rather than detection.

## Methods

### Study Design

A quasi-experimental design was piloted to evaluate ChatGPT’s performance on a range of assessment types, comparing its results with submitted assessments of past students. This exploratory approach was intended to provide preliminary insights and inform the design of larger-scale studies. While similar methods have been applied in ChatGPT response evaluation within the education sector, this study pilots the approach in the context of DIGHIM, where it has not yet been examined [11,16,41].

### Assessment Materials and Markers (Participants)

Assessments from 5 DIGHIM subjects were purposively selected because of specific assessment characteristics, to maximize diversity and breadth in the type and nature of assessments chosen (Table 1). Past students’ deidentified assessments, originally submitted for academic credit, were reevaluated for research purposes as a benchmark against which the ChatGPT-generated responses were compared. Markers, blinded to the origin of each assessment, were responsible for evaluating both the ChatGPT-generated assessments and the past students’ assessments. Markers were academic staff, both full-time and casual, with postgraduate qualifications and advanced professional and teaching experience in DH or HIM relevant to the specific assessments they were assigned, and with demonstrated expertise in evaluating student work using standardized marking rubrics.

**Table 1:** Summary of assessment types/sample chosen for the study.

Assessment type	Level	Discipline	Assessment description	Experimental (AI^a^) group assessments marked, n	Control (student) group assessments marked, n
Digital health solution design	Masters	Digital health	Propose and justify a structured approach to addressing a specific health care challenge through a digital health solution, incorporating design principles, implementation frameworks, and the consideration of barriers and enablers.	4	3
Digital health business case proposal report	Masters	Digital health	Develop a proposal identifying gaps in a digital health system, analyzing barriers, and recommending evidence-based, innovative solutions.	4	3
Health information management reflective assessment	Fourth-year undergraduate	Health information management	Reflection on health information management students’ professional practice (work-integrated learning) placement experience and learning	4	3
Health database SQL programming	Second-year undergraduate	Health information management	Use of SQL to query and analyze a health database, generating reports, and extracting relevant data	4	3
HIM^c^ health classification online quiz examination	First-year undergraduate	Health information management	Online examination comprising long, short, and objective (MCQ^b^, true/false, and 1-word answer) questions, assessing knowledge of health classification systems (*ICD-10-AM*^d^) and clinical coding principles	2	3

^a^AI: artificial intelligence.

^b^MCQ: multiple-choice question.

^c^HIM: health information management.

^d^*ICD-10-AM*: International Classification of Diseases, Tenth Revision, Australian Modification.

### Sample

#### Control Group

Subject coordinators selected a sample of student assessments (3 per each assessment type) completed between July 1, 2023, and June 30, 2024, to form the control group. To ensure representativeness, assessments were chosen across 3 grade bands: high performance (>80%), medium performance (70%-79%), and low performance (50%-59%). This resulted in the inclusion of a total of 15 student assessments across the 5 subjects and 5 assessment types. All assessments were deidentified prior to analysis to maintain student anonymity.

#### Experimental Group

ChatGPT was used to complete the same assessment tasks as were selected for the control group. A total of 2-4 AI-generated assessments were created for each assessment type (Table 1; n=18) to reflect variations in how a typical student might approach the task. The criteria for these variations and their alignment with student input patterns are detailed below in the Experiment section.

In total, 33 assessments, across the 5 assessment types, were selected for the study: 18 were AI-generated (experimental group), and 15 were past students’ assessments (control group). The 5 assessment types include both undergraduate and postgraduate assessments, reflecting core educational activities that develop competencies directly relevant to medical education, including clinical documentation, health classification, health data analysis, DH system design, and reflective practice in health care settings.

### Experiment

#### Overview

**Figure 1:**
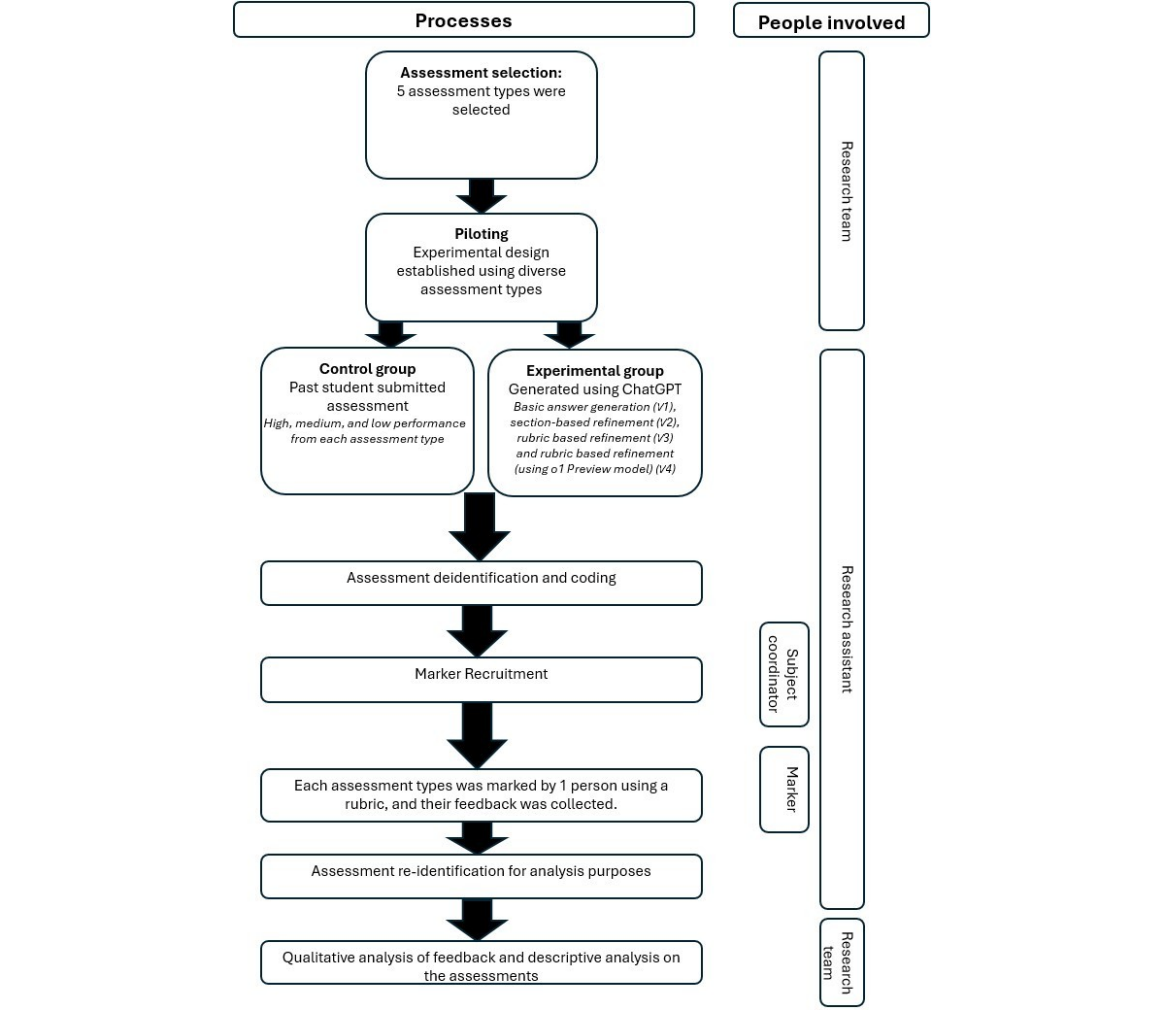
Overview of methodology.

An independent research assistant (RA) followed a structured process, established by the researchers, to gather ChatGPT assessment responses for the chosen sample of assessments (Figure 1).

#### Generating ChatGPT Responses

To ensure reliability, completeness, and alignment with academic expectations, multiple submission versions (ie, basic answer generation [V1], section-based refinement [V2], rubric-based refinement [V3], and rubric-based refinement using o1 Preview model [V4]) of assessment instructions were developed for each assessment type for submission to ChatGPT and its versions (Table 2). Following the preliminary testing phase described in the Testing and Review subsection below, a refined and detailed protocol was created to guide the research assistant in generating all 4 structured versions (V1-V4; Multimedia Appendix 1).

It was expected that full-instruction (V1) and step-by-step approaches (V2 and V3) would allow for a nuanced analysis of AI-generated outputs, with V2 and V3 specifically designed to assess how incremental guidance impacted response quality. Furthermore, inclusion of rubrics (V3) was expected to further improve contextual alignment. The inclusion of o1 Preview (V4) used the o1 model, which was designed for advanced reasoning, and provided insights into GenAI’s evolving capabilities. For SQL tasks, submission versions (V2 and V3) included the database schema and rubric to support contextual accuracy; however, the underlying health dataset itself was not provided to ChatGPT. In contrast, for objective-style questions in the health classification assessment, only 2 submission versions were needed due to the structured format of the questions and the straightforward nature of the required responses. It is important to note that in all versions, ChatGPT was provided with the same assessment instructions and marking rubrics that were originally given to students, ensuring comparability between human and AI-generated work.

**Table 2:** Description of artificial intelligence–generated submission versions and prompting strategies.

Assessment type and submission version		Description
**Subjective and analytical assessments (reflective assessment, business case proposal/case study, and digital health solution)**		
	V1	Full assessment instructions entered into ChatGPT using the GPT-4 model for a single-step response generation.
	V2	Assessment broken into parts as per instructions; ChatGPT using the GPT-4 model generated each part separately and compiled.
	V3	Same as V2, with the rubric provided to guide response generation for better alignment with criteria.
	V4	Same as V3, but generated using the o1 Preview model to evaluate differences in performance.
**SQL/programming assessment**		
	V1	Query instructions provided without a schema for response generation using GPT-4.
	V2	Query instructions with the schema provided to enhance contextual accuracy using GPT-4.
	V3	Schema and rubric provided to ensure responses aligned with evaluation criteria using GPT-4.
	V4	Same as V3, but generated using the o1 Preview model for comparative analysis.
**HIM health classification/clinical coding online quiz examination (scenario-based and objective questions)**		
	V1	Objective questions generated using GPT-4.
	V2	Objective questions generated using the o1 Preview model to compare differences in outputs.

#### Blinded Review Preparation

The research assistant formatted ChatGPT-generated responses to resemble student submissions. These AI-generated responses were then mixed with real, deidentified student assessments to maintain blinding. To ensure an unbiased review process, all assessments were anonymized and assigned coded identifiers.

#### Assessment Allocation

Based on their expertise in the subject, each subject coordinator suggested potential markers to the RA to assess and mark both ChatGPT responses and student assessments. The RA contacted potential markers individually via email to seek their participation and to shortlist 1 marker per assessment type. Markers were provided with participant information consent forms. To maintain impartiality, the nominated markers did not include the academics who originally marked the student assessments. Assessments were randomly assigned to the markers by the RA, acting as an independent coordinator to ensure a fair distribution and prevent any conflicts of interest.

#### Marking Process

Markers followed a standardized marking rubric to evaluate the assessments. To ensure consistency in grading, clear instructions were provided by the principal investigator and communicated by the independent RA. Additionally, support was available throughout the process to address any questions or uncertainties. In addition to numerical scores, markers provided written feedback aligned with the rubric criteria, along with overall free-form comments to justify their grading and offer insights into the quality of the work. To further ensure reliability, if a discrepancy of more than 10 marks was identified in any student assessment (between the original mark and remark), all assessments within that assessment type were independently reviewed by a second subject expert. Any differences were then discussed, and a consensus was reached to finalize the grades.

#### Data Tracking and Management

The RA maintained a Microsoft Excel spreadsheet to track the marking process, including details of assessments sent to staff members and their feedback.

#### Testing and Review

As part of the experimental process, an initial round of testing was undertaken to refine the methodology and confirm the feasibility of the planned research. This involved a smaller sample of assessments across various types (Table 2) to trial the generation of AI responses, the collection and deidentification of student work, and the blinded review process (see the sections “Blinded Review Preparation,” “Assessment Allocation,” “Marking Process,” and “Data Tracking and Management” above). Insights from this stage were used to make necessary adjustments prior to the main experiment. The assessments used during this testing phase were excluded from the final data analysis. During preliminary testing, we determined that generating multiple outputs from the same input was not suitable for this study’s design. When repeated prompts were issued within the same ChatGPT account, model-state carryover effects created dependency between responses due to latent conversational memory, thereby compromising independence across outputs. Conversely, when the same inputs were generated using different accounts, the resulting outputs were highly similar in structure, sequencing, and wording. Such similarity posed a risk to the blinded marking process, as a single marker evaluating near-identical responses could readily identify them as AI-generated, introducing potential confirmation bias. For these reasons, we adopted a structured versioning approach (V1-V4) instead of generating multiple outputs for identical inputs.

### Synthesis and Analysis

Marks and qualitative feedback provided by academic markers were compiled for each assessment type. Descriptive statistics (means, percentages, and score ranges) were calculated across all student grade bands (high, medium, and low) to support the blinded marking process and ensure natural variation within the control group. While ChatGPT outputs were compared against all remarked student assessments, the primary analytical focus was on the highest-graded submissions, as these offered a stable and meaningful benchmark for assessing whether AI-generated work could approximate high-quality human performance. Performance was examined both within individual assessment types and across assessment categories.

Comparative trend analysis was undertaken to evaluate progression across ChatGPT versions (V1-V4), focusing on improvements associated with structured prompting, schema inclusion, and rubric integration. In addition, rubric-level synthesis was conducted to identify recurring strengths and weaknesses.

Feedback comments for both student and AI-generated submissions were reviewed and compared, with particular focus on top-performing student work. This comparison helped identify where the evaluative feedback on AI outputs differed most noticeably from that of human-authored work.

Results were further synthesized into cross-assessment comparisons, with performance grouped into broader task categories (objective, reflective, descriptive analytical, scenario-based analytical, communication/referencing, and programming) to identify where AI demonstrated relative strengths versus persistent limitations.

### GenAI Tools Used

The study used GPT-4 (free version/limited) and o1 Preview (pro/paid version) for the experimentation. The researchers opted out of ChatGPT’s AI training model using an available feature, ensuring that the submitted assessment guidelines and instructions were not used to further train the AI. All necessary privacy settings were enabled to maintain data confidentiality throughout the research process.

### Ethical Considerations

The research study was approved by La Trobe University’s Human Research Ethics Committee (application number HEC24286). No student assessment submissions, student-generated content, or identifiable educational data were entered into any GenAI tools as part of this study. Only publicly available assessment instructions and marking rubrics were provided to ChatGPT to generate AI responses. All student work used for comparison was deidentified, stored securely, and assessed offline by academic markers.

## Results

### Performance by Assessment Type

#### Overview

This section provides a detailed breakdown of ChatGPT’s performance across each assessment type, with comparisons to the highest-graded student assessments. Comprehensive data analysis and full qualitative feedback for all assessments are provided in Multimedia Appendix 2. This appendix includes comparisons with all types of student submissions. Results are presented by assessment type and task category in the same sequence as outlined in the Methods section, enabling direct comparison between study design and outcomes.

#### Assessment Type 1: DH Solution Design

This assessment required students to design and justify a DH solution using implementation frameworks and a critical analysis of barriers and enablers, assessed against rubric criteria for problem framing, contextual relevance, and professional communication.

AI-generated responses showed progressive improvements with structured prompting and rubric integration, most notably in GPT-4 (V3), which scored 69% compared to 56% for GPT-4 (V1; Table 3). Overall, even with these refinements, AI outputs lacked the depth, specificity, and contextual alignment observed in student submissions. Notably, the use of the more advanced o1 model (V4) did not improve performance over GPT-4 (V3), scoring only 63%, and continued to exhibit generic recommendations and limited tailoring to the scenario. In contrast, the top-performing student submission (81%) demonstrated nuanced justification, visual clarity, and strong alignment with project objectives.

**Table 3:** Comparison of outcomes from ChatGPT submission versions for digital health solution assessment.

GPT model and submission version	Score (%)	Key positive points noted by the marker	Key points for improvement noted by the marker
GPT-4 (V1^a^)	56	Design stages justification incomplete	Vague barrier discussion Unclear language Misplaced citations
GPT-4 (V2^b^)	60	Improved clarity and referencing (than V1) Better articulation of barriers	Generic roadmap activities Limited alignment with goals
GPT-4 (V3^c^)	69	Design approach supported with clear evidence Better understanding of challenges Strong structure Clearer communication Improved referencing	Activities remained broad Lacked visual aids and specificity
o1 Preview (V4^d^)	63	Consistent framework discussion Professional tone	Roadmap remained generic Limited contextual alignment

^a^V1: basic answer generation.

^b^V2: section-based refinement.

^c^V3: rubric-based refinement.

^d^V4: rubric-based refinement using o1 Preview model.

#### Assessment Type 2: DH Business Case Proposal Report

This assessment required students to develop a DH business proposal identifying system gaps and recommending innovative technologies, with emphasis on evidence-based justification, analysis of implementation barriers, and professional communication demonstrating health care value and impact.

ChatGPT-generated submissions yielded similar scores across versions (Table 4). GPT-4 (V2) scored the highest (68%), showing improved referencing and clearer structure. However, despite incremental refinements, all AI outputs were critiqued for their generic content, lack of tailored insights, and absence of visual elements such as patient journey maps or system flow diagrams. Notably, the more advanced o1 Preview model (V4) did not improve performance (score: 64%) and continued to present directive rather than strategic framing. In contrast, the top-performing student submission (score: 82%) demonstrated personalized problem-solution alignment, stronger analytical depth, and enhanced clarity through well-integrated visual aids.

**Table 4:** Comparison of outcomes from ChatGPT submission versions (V1-V4)—digital health business proposal/case study assessment.

GPT model and submission version	Score (%)	Key positive points noted by the marker	Key points for improvement noted by the marker
GPT-4 (V1^a^)	62	Basic identification of system gaps logical structure	Weak justification of solutions insufficient references vague insights
GPT-4 (V2^b^)	68	Clearer articulation of barriers improved referencing and layout	Lacked personalization limited strategic alignment with health care needs
GPT-4 (V3^c^)	67	Stronger evidence-based rationale professional tone	Solutions not well integrated with patient needs absence of visual aids such as journey maps
o1 Preview (V4^d^)	64	Consistently identified issues and proposed digital interventions	Lacked strategic framing weak linkage between problems and proposed actions

^a^V1: basic answer generation.

^b^V2: section-based refinement.

^c^V3: rubric-based refinement.

^d^V4: rubric-based refinement using o1 Preview model.

#### Assessment Type 3: HIM-Reflective Assessment

This assessment required students to critically reflect on their HIM placement, using empirical evidence to evaluate professional competencies, identify strengths and weaknesses, set actionable development goals, and demonstrate professional communication.

ChatGPT-generated responses showed a wide range in performance (Table 5). GPT-4 (V3) achieved the highest AI score (80%), presenting a well-structured report with detailed reflections and systematic use of empirical evidence, even surpassing the top student marked (70%). However, it exceeded the word limit and lacked visual enhancements, while some goals remained broad and misaligned with competencies expected in the health industry. Earlier versions showed key weaknesses: V1 (53%) was concise but underdeveloped and lacked depth in reflection and linkage to placement experience, and V2 (70%) improved on structure and relevance but included fabricated references and overly clinical goals. The most advanced version, o1 Preview (V4), scored only 55%, meeting word limits but offering shallow insights and repetitive, vague reflections.

While ChatGPT demonstrated fluency, structure, and effective use of reflective frameworks, it fell short in personalization, contextual relevance, and depth, elements that distinguished the top student submission, which provided clear placement-specific insights, nuanced analysis, and realistic strategies for professional development. This also aided in evaluating whether AI could produce realistic reflective narratives and to inform how such assessments might be strengthened in the future.

**Table 5:** Comparison of outcomes from ChatGPT submission versions (V1-V4)—HIM reflective assessment.

GPT model and submission version	Score (%)	Key positive points noted by the marker	Key points for improvement noted by the marker
GPT-4 (V1^a^)	53	Structured format Identified gaps in reflective practice	Omitted details Weak placement linkage Lacked depth and clarity
GPT-4 (V2^b^)	70	Strong organization Good competency linkage, visual structure	Overly clinical focus Exceeded word count Included fabricated reference
GPT-4 (V3^c^)	80	Comprehensive, well-referenced Detailed placement reflection	Wordy Lacked visuals Some goals too broad for HIM context
o1 Preview (V4^d^)	55	Concise and grammatically sound Some relevant evidence	Shallow reflection Repetitive content Weak critical analysis

^a^V1: basic answer generation.

^b^V2: section-based refinement.

^c^V3: rubric-based refinement.

^d^V4: rubric-based refinement using o1 Preview model.

#### Assessment Type 4: Health Database SQL Programming

This assessment evaluated students’ ability to assess identification of health information needs, write accurate SQL queries to analyze health data, extract relevant information, and interpret results to support health care decision‑making.

AI-generated responses demonstrated only modest improvement across iterations, with schema and rubric integration resulting in higher scores (Table 6). GPT-4 (V1), generated without schema input, scored the lowest (17%), with all queries based on incorrect table and column names. Schema inclusion in V2 improved structural accuracy (47%), but errors in data grouping and missing outputs persisted. V3 (49%) refined query logic slightly, though misspellings and omission of key fields remained problematic. The most advanced model, o1 Preview (V4), achieved the highest AI score (56%), correctly executing about half of the queries and demonstrating improved adherence to SQL conventions, but still suffered from inaccuracies in age group breakdowns, incorrect counts, and missing contextual details such as diagnosis descriptions.

In contrast, the top student submission (100%) showed precise application of SQL logic, full query completion, and context-aware interpretation of health trends. It demonstrated superior attention to schema structure, data accuracy, and professional communication in presenting findings, areas where ChatGPT consistently underperformed.

**Table 6:** Comparison of outcomes from ChatGPT submission versions (V1-V4)—health database SQL programming.

GPT model and submission version	Score (%)	Key positive points noted by the marker	Key points for improvement noted by the marker
GPT-4 (V1^a^)	17	Correct use of SQL structure Basic understanding of syntax	All table/column names incorrect No valid outputs Missing queries
GPT-4 (V2^b^)	47	Some correct queries Schema improved the structural logic	Wrong grouping Spelling errors Partial outputs Inconsistent results
GPT-4 (V3^c^)	49	Better rubric alignment Clearer structure	Inaccurate grouping Incomplete queries Diagnosis data omitted
o1 Preview (V4^d^)	56	Improved accuracy Half the queries returned correct results	Ongoing errors in counts and terminology Limited interpretation

^a^V1: basic answer generation.

^b^V2: section-based refinement.

^c^V3: rubric-based refinement.

^d^V4: rubric-based refinement using o1 Preview model.

#### Assessment Type 5: HIM Health Classification/Clinical Coding Online Quiz Examination

This quiz assessed students’ application of *ICD-10-AM* (*International Classification of Diseases, Tenth Revision, Australian Modification*) health classification standards through scenario-based coding, line coding, and objective questions.

AI responses (GPT-4 [V1] and o1 Preview [V2]) performed best in the objective section (88%) of the quiz but scored only 7% in scenario-based coding tasks, with a 32% overall score, compared to 87% scored by the top student (Table 7). Key issues included incorrect block numbers, inaccurate sequencing, and vague or missing code justifications. In contrast, the top student demonstrated precision and contextual understanding aligned with national coding standards. These results reinforce that while AI handles structured recall effectively, it lacks the nuanced reasoning required for applied clinical coding.

**Table 7:** Comparison of outcomes from ChatGPT submission versions (V1-V4)—health classification quiz. Only 2 versions were tested, as rubric- and breakdown-based versions were not applicable to this quiz format.

GPT model and submission version	Score (%)	Key positive points noted by the marker	Key points for improvement noted by the marker
GPT-4 (V1^a^)	32.4	Strong performance in the objective section (14/16 correct)	Incorrect block numbers Poor sequencing Inaccurate tabular usage
o1 Preview (V2^b^)	32.4	Consistent objective accuracy Good understanding of conventions	Weak in scenario coding Missing procedural codes Vague codes/justifications

^a^V1: basic answer generation.

^b^V2: section-based refinement using o1 Preview model.

### Cumulative Performance Comparison Across Assessment Types and Tasks

#### Comparative Analysis Across Assessment Types

**Figure 2:**
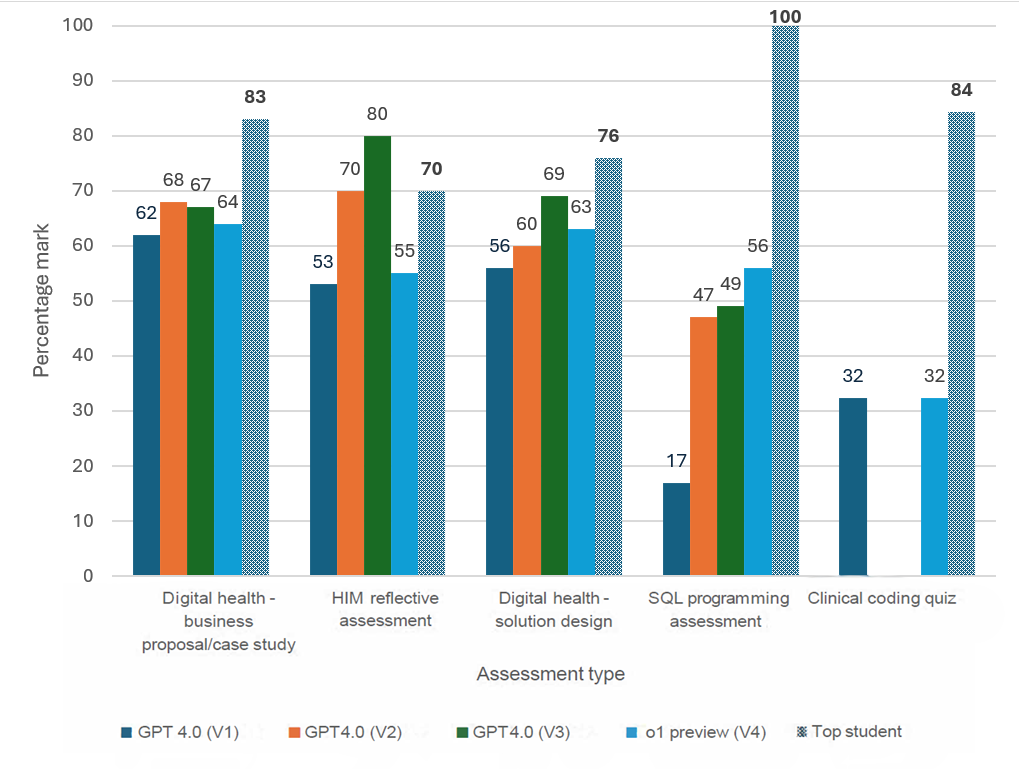
Overall performance comparison across assessment types. V1: basic answer generation; V2: section-based refinement; V3: rubric-based refinement; V4: rubric-based refinement using o1 Preview model.

An overall performance comparison across different versions of ChatGPT responses, based on average scores, revealed noticeable variations in performance across assessments (Figure 2).

Descriptive and reflective assessments consistently achieved higher average scores in ChatGPT-generated responses, compared to technical assessments such as SQL programming and health classification quiz, which received substantially lower average scores. Lower averages in these tasks reflected the complexity of accurately interpreting technical prompts, adhering to database structures, and executing precise scripts. Progression from V1 to V4 in technical tasks demonstrated the importance of context, schema inclusion, and rubrics, which helped improve performance over iterations. However, even the best-performing versions did not match the precision and depth required for these tasks, underlining limitations in handling computational assessments.

Overall, V3 achieved the highest average scores across assessment types, benefiting from rubric-driven structuring and better alignment with task criteria. V2 showed notable success in descriptive tasks due to its structured approach, while V4 demonstrated improvements in technical assessments through contextual and schema support. In contrast, V1 consistently underperformed across most assessments, reflecting its reliance on single-step, unguided generation, which often lacked depth, accuracy, and alignment with task expectations.

#### Comparative Analysis of ChatGPT Versions Across Task Types

**Figure 3:**
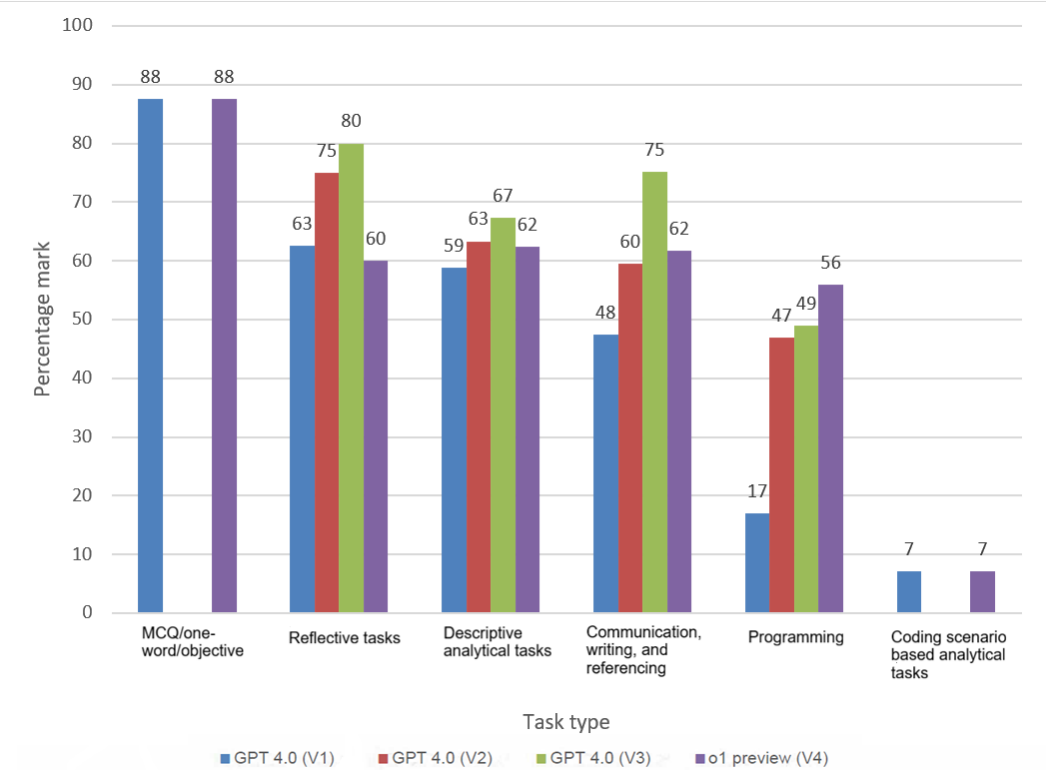
Overall performance comparison of ChatGPT versions across assessment task types/rubric criteria. V1: basic answer generation; V2: section-based refinement; V3: rubric-based refinement; V4: rubric-based refinement using o1 Preview model.

Evaluating ChatGPT’s performance across assessment task types, ChatGPT performed best on objective tasks, showing strong accuracy in factual, rule-based questions. Reflective tasks followed, with later versions demonstrating improved reasoning. Descriptive analytical tasks were next in terms of average performance, though gains plateaued in newer versions. Communication and referencing tasks also showed similar performance with a clearer structure in later iterations. Programming tasks improved over time but continued to face challenges in accuracy and contextual understanding. The lowest performance was in complex scenario-based health classification tasks, reflecting current limitations in contextual and analytical reasoning (Figure 3).

## Discussion

### Overview

This research aimed to pilot the evaluation of ChatGPT’s performance across diverse DH and HIM assessment tasks, with a focus on promoting academic integrity. This analysis not only revealed the specific contexts in which ChatGPT excelled or fell short but also suggested broader implications for designing assessments that foster critical thinking, ethical practice, and real-world applicability. By situating ChatGPT’s performance within the study’s original goals, we were able to gain clearer insights into the potential and the limitations of GenAI in academic and professional environments. However, because this pilot examined only the final outputs generated by ChatGPT, it does not evaluate AI’s role as a formative learning partner, such as when students iteratively critique, refine, or codevelop AI-generated drafts.

### Principal Findings and Comparison With Prior Literature

The DH case study and solution design assessments revealed clear limitations in ChatGPT’s capacity to address complex, context-specific problems. While AI-generated submissions consistently identified foundational frameworks and produced well-structured outputs, they lacked the depth and contextualization required to develop effective, patient-specific solutions. Weak alignment between identified gaps and proposed interventions reflects broader challenges in maintaining contextual coherence. This is consistent with prior findings across disciplines that report similar shortcomings despite fluent language, structural coherence, and high grammatical accuracy [16,44]. The absence of visual artifacts, such as patient journey maps or flowcharts, further constrained the communication of complex ideas, underscoring limitations in GenAI’s ability to produce meaningful visual representations [45]. These findings reinforce the essential role of human expertise in refining and contextualizing solutions. For DIGHIM assessments, tasks should prioritize deep contextual engagement, patient-specific scenarios, and dynamic problem-solving. Requiring the integration of real-world data, graphical visualizations, and detailed case-based justification can better assess applied competence while reducing overreliance on AI tools. This approach aligns with constructivist learning theory, which emphasizes learning through authentic engagement rather than surface-level reproduction [46,47], and with higher-order cognitive processes in Bloom taxonomy that current GenAI systems cannot reliably replicate [48,49].

Reflective assessments demonstrated ChatGPT’s strongest performance, with later versions, particularly GPT-4 (V3), achieving the highest score at 80% and surpassing student submissions in structure, coherence, and adherence to reflective frameworks. Similar patterns have been reported in dental education, where ChatGPT performed strongly in structured-portfolio tasks [17]. However, despite polished language and systematic organization, AI-generated reflections often lacked genuine depth, contextual nuance, and personalized insight, which are central to HIM professional development. These results indicate a need to redesign reflective assessments to preserve authenticity. This aligns with social cognitive theory, which emphasizes authentic self-reflection, mastery experiences, and the development of self-efficacy as central to professional learning, all of which are elements that GenAI cannot meaningfully replicate [50]. Emphasizing personalized insights, unique contextual connections, and critical evaluation of lived experiences, alongside dynamic scenarios, peer interaction, or real-time contextual observations, can reduce AI dependence while reinforcing academic integrity and professional skill development [51,52].

Objective assessments, including multiple-choice and true or false questions, showed consistently high AI performance with minimal error rates. This reflects known strengths of AI in structured, rule-based tasks requiring factual recall and logical reasoning and aligns with prior evaluations of GenAI performance on objective assessments [53-56]. However, this raises concerns regarding the validity of such tasks in measuring individual learning, particularly in online contexts. To maintain assessment integrity, objective questions should be supplemented with higher-order components such as justification, explanation, or reasoning. Embedding real-world context and multistep problem-solving can further enhance authenticity and alignment with learning outcomes while limiting AI dominance [57].

Clinical coding scenario-based assessments revealed a clear performance gap between AI and students, particularly in tasks requiring nuanced contextual interpretation and application of coding rules. While AI performed comparably on objective components, it struggled with complex scenarios, resulting in significantly lower scores than high-performing students. This aligns with US-based findings showing that ChatGPT performs adequately for simple, single-diagnosis coding tasks but struggles with complex patient data [58,59]. Soroush et al [59] reported that GPT-4 achieved only a 34% exact match rate for *ICD-10-CM* (*International Classification of Diseases, Tenth Revision, Clinical Modification*)codes and concluded that LLMs lack a complete internal representation of medical coding rules, rendering them unsuitable for clinical coding tasks. These findings underscore the need for assessments that emphasize contextual reasoning and real-world coding application, reinforcing the importance of developing independent clinical coding proficiency.

SQL programming assessments highlighted further limitations in ChatGPT’s ability to perform technically precise, context-dependent tasks. Although later versions showed incremental improvement, persistent issues included incorrect table and column usage, overreliance on prompts, limited understanding of health data conventions, and failure to execute complex multistep queries. Errors such as misspellings and the absence of real-time testing and debugging capabilities further undermined output reliability. These findings contrast with prior research reporting strong SQL generation performance by LLMs [60], suggesting that health database contexts demand deeper domain knowledge and contextual awareness. Previous work has emphasized the importance of iterative testing and contextual understanding in SQL code generation [61]. For educators, this highlights the value of assessments that extend beyond syntax to include debugging, real-world application, and analytical interpretation of health data, thereby fostering job-ready competencies that AI cannot fully replicate.

In communication, writing, and referencing tasks, ChatGPT demonstrated strong performance in structure, clarity, and adherence to academic conventions, particularly in later versions such as V3. Nonetheless, limitations in depth, contextual relevance, repetition, and critical engagement persisted, especially in longer writing tasks. Similar findings have been reported in business education, where AI-generated responses for extended academic writing scored between 40% and 77% due to insufficient depth and analysis despite high grammatical quality [16]. Issues such as hallucinated references and inconsistent citation formatting further undermined credibility, consistent with prior research [62,63]. These results indicate that while AI can support foundational academic writing, it struggles with nuanced argumentation and original critical thought. Assessment designs should therefore require critical engagement with sources, precise referencing, and personalized application of concepts to ensure authentic student learning.

Chain prompting, involving iterative refinement of prompts, significantly improved AI performance, particularly in descriptive and reflective tasks, with later versions benefiting from rubric-aligned guidance. Comparable improvements have been observed in pharmacy education, where structured prompting enhanced performance in knowledge recall tasks [56]. This demonstrates the effectiveness of multistep prompting in optimizing AI outputs for well-defined assessment formats.

Overall, o1 Preview (V4) underperformed in descriptive tasks relative to V3, although performance was comparable or improved in simpler objective assessments. This suggests that newer AI versions do not necessarily yield better outcomes for complex, context-driven tasks. While earlier studies reported GPT-4 superiority in medical licensing examinations [64] and basic sciences [65], such improvements were not consistently observed in this study. There was no clear evidence that o1 Preview enhanced performance in tasks requiring critical thinking or reflection. These findings highlight the importance of assessment designs that prioritize originality, contextual understanding, and higher-order cognition over tasks that can be easily completed using AI assistance.

### Strengths and Limitations

The findings from this pilot study highlight the importance of designing assessments that align with the evolving roles in HIM and DH while fostering the ethical and practical use of GenAI. Health information managers play a crucial role in health data management, analytics, and health information and communication technology [33], and as DH increasingly integrates disciplines such as clinical care, data science, and IT [66], it is essential for educational assessments to reinforce interdisciplinary skills that align with the evolving demands of these domains [67,68]. Recent evidence highlights that traditional assessment methods in higher education are increasingly ineffective in GenAI-facilitated learning environments, necessitating redesigned assessments that promote critical thinking, creativity, and lifelong learning skills [28]. For instance, practical redesign approaches, such as “flipped assessment,” can be implemented, where students cocreate and then critically validate AI-generated items under educator supervision, improving confidence while maintaining expert oversight [31]. This type of redesign aligns with this study’s recommendation for tasks that encourage students to critique, refine, and contextualize AI outputs rather than merely generate them.

Assessments should emphasize tasks that require contextual understanding, technical proficiency, and decision-making. Furthermore, these assessments should integrate AI as a tool to augment, rather than a substitute for critical thinking. For example, students could refine AI-generated clinical coding or SQL outputs to align with standards-based frameworks, critically evaluate AI-generated business proposals for depth and contextual relevance, or integrate visualizations such as patient journey maps into health information reports. These approaches not only build essential competencies such as critical analysis, programming, and communication but also encourage students to navigate AI’s limitations and responsibly incorporate its outputs into their work, promoting the ethical use of GenAI to support, rather than replace, student efforts.

In DIGHIM education, AI use in assessments should be carefully structured to align with professional competencies while maintaining academic integrity. Some tasks should avoid reliance on AI, particularly those requiring personal reflections, ethical decision-making, and critical thinking in complex DH scenarios. Other tasks can adopt AI for efficiency, such as assisting in summarizing health policies, generating structured reports, or organizing data-driven insights. Finally, AI-generated content can be adapted in assessments that require students to critically refine, validate, and contextualize AI outputs, such as evaluating AI-generated clinical coding recommendations, refining SQL queries for health databases, or assessing the applicability of AI-generated DH solutions within industry frameworks. Additionally, educators and students must ensure privacy by refraining from supplying GenAI tools with copyrighted or sensitive health data and should receive structured training on the responsible and ethical use of AI in DH and HIM to support professional readiness.

Taken together, current 2024-2025 evidence converges on a dual imperative: redesign assessments to cultivate critique, verification, and reflection skills, and establish clear governance mechanisms, including policy guidance, training programs, and disclosure norms, proportionate to the evolving capabilities and risks of GenAI systems [31,32,38].

These approaches prepare students for real-world applications of GenAI in HIM and DH roles, where such tools may be used to assist with technical tasks such as data analysis, report generation, and strategic planning. By designing assessments that both test and build skills in these areas while fostering responsible AI use, educators can equip graduates to navigate the interdisciplinary and technology-driven landscape of modern DH care effectively.

Table 8 provides a summary of important recommendations.

This study had several limitations. First, as a pilot study, the findings are preliminary and intended to provide directional insights rather than definitive conclusions; however, the use of multiple assessment types and structured AI submission versions was intended to strengthen internal comparison. Larger-scale studies are needed to confirm these patterns. Second, the quasi-experimental design lacked random assignment, which may limit generalizability. This was partially mitigated by comparing AI-generated submissions with authentic, previously graded student work under blinded conditions. Future studies could strengthen external validity through multiinstitutional or randomized designs. Third, assessments were marked by discipline-specific academic staff, and individual grading variability may have influenced results despite mitigation strategies, including standardized rubrics, blinded review, and second-marker consensus when remarked scores differed by more than 10 marks. Future work could further enhance reliability by incorporating multiple independent markers per assessment. Fourth, the number of assessment samples within each category was limited, which may reduce the robustness of task-specific conclusions. This was addressed by selecting assessments across multiple levels and disciplines, though larger samples are required in future studies. Fifth, the study could not fully replicate real-time student interactions with GenAI, such as iterative prompting or blended human-AI workflows. The structured versioning approach (V1-V4) was used to approximate common usage patterns, but longitudinal studies are needed to capture authentic use over time. Sixth, while all student submissions underwent formal academic integrity checks and no concerns were identified, it was not possible to determine with absolute certainty whether any student work involved AI assistance, which may have influenced comparisons. Finally, the study did not systematically assess the detectability of AI-generated content. Although markers occasionally inferred AI authorship, these judgments were informal and sometimes inaccurate, consistent with prior reports of false positives [16,30,69] and highlighting the unreliability of human detection methods.

Future research should explicitly investigate the accuracy of markers in distinguishing AI- from student-generated work and consider how assessment design can emphasize originality, critical thinking, and personalized insights to reduce reliance on detection and strengthen academic integrity. Another area that future studies could explore is expanding the scope beyond ChatGPT to include other advanced LLMs (eg, Gemini, Claude, and AI-assisted coding tools such as GitHub Copilot), to allow broader comparison of capabilities and provide a more comprehensive understanding of how different AI systems perform across assessment types in DIGHIM.

**Table 8:** Recommendations for ethical assessment design in Digital Health and Health Information Management in the artificial intelligence (AI) era.

Theme	Key findings	Recommendations
Contextual and higher-order assessment design	AI struggled with context-specific, patient-centered, and judgement-based tasks. Underperformance in descriptive, analytical, and coding scenario questions.	Use assessments requiring deep contextualization, scenario complexity, and justification of decisions. Emphasize ethical reasoning, critique, and higher-order application rather than factual recall.
Reflective and authentic assessments	AI produced a strong structure but fabricated plausible experiences. Risk to authenticity in reflective tasks.	Require personalized and context-verified reflections (eg, workplace events, supervisor-signed logs). Use scenario-based reflections, peer engagement, or real-time journaling to ensure authenticity.
Objective and procedural tasks	AI performed strongly on MCQs^a^, true/false, and structured objective items. Risk of undermining originality.	Add reasoning, explanation, or linked vignettes to objective tasks. Combine correctness with justification to assess understanding.
Technical and programming tasks (SQL/coding)	AI struggled with schema use, debugging, and domain conventions. Frequent inaccuracies despite rubric/schema input.	Use live, hands-on coding tasks requiring testing, debugging, and scenario-based logic. Assess understanding of health-data structures and domain-specific conventions.
Academic writing and referencing	Strong structure but limited depth and contextual argumentation	Require deeper analysis, original reasoning, and evidence validation Include tasks where students examine or verify AI-generated claims.
Ethics, equity, privacy, and governance	Prompting skill differences create inequities AI detection unreliable Privacy and rubric transparency concerns.	Provide standardized AI literacy training Avoid overreliance on detection tools; design for originality and personal insight Train students not to enter sensitive data into AI tools. Use rubrics that guide learning without enabling “AI gaming.” Develop institutional policies for ethical AI use.

^a^MCQ: multiple-choice question.

### Conclusions

This pilot study has highlighted the potential and limitations of ChatGPT in HIM and DH assessments. While GenAI demonstrates strengths in structured tasks and foundational content generation, it struggles with contextualization, depth, and the technical precision required for assessments requiring independent critical thinking. These findings emphasize the need for carefully designed assessments that integrate AI ethically and prioritize tasks that require human judgment and contextual understanding to ensure meaningful learning outcomes and academic integrity.

## Appendix

Multimedia Appendix 1 Protocol for research assistant.

Multimedia Appendix 2 Detailed performance analysis by assessment type.

## Abbreviations

AI: artificial intelligence
DH: digital health
DIGHIM: Digital Health and Health Information Management
GenAI: generative artificial intelligence
HIM: health information management
ICD-10-AM: International Classification of Diseases, Tenth Revision, Australian Modification
ICD-10-CM: International Classification of Diseases, Tenth Revision, Clinical Modification
LLM: large language model
RA: research assistant
V1: basic answer generation
V2: section-based refinement
V3: rubric-based refinement
V4: rubric-based refinement using o1 Preview model

## Data Availability

Summary data and detailed analyses supporting this study are provided in the manuscript and Multimedia Appendix 2. Access to underlying deidentified student assessment data is restricted due to ethical and privacy considerations; however, reasonable requests for additional deidentified data may be considered by the corresponding author, subject to institutional ethics approval.
