# Supplementary material for: Susceptibility of Assessment Types to AI-Generated Content in Digital Health and Health Information Management Education: Quasi-Experimental Pilot Study
Source: JMIR Med Educ. 2026 Mar 30;12:e82988. doi: 10.2196/82988 (PMC13077276; doi:10.2196/82988)
Supplement: Multimedia Appendix 1 [file mededu_v12i1e82988_app1.docx]

# ****Protocol sent to RA for generating ChatGPT responses****

## Introduction

You will be instructed to generate four different variations in the quality of answers from a single assessment instruction using generative AI (ChatGPT). We will be utilizing different levels of **chain prompting techniques (quality 1-4)** to guide the AI in producing responses of varying quality. This method helps in achieving different levels of depth, detail, and alignment with grading rubrics, providing a range of outputs for comparison or submission enhancement.

## Protocol (Quality 1-3)

## Step 1: Log In to ChatGPT Premium

1. **Visit the ChatGPT Website**
   - Open your web browser and navigate to [chat.openai.com](https://chat.openai.com).
2. **Log In to Your Account**
   - Click on the **"Log In"** button.
   - Enter following **email address** and **password**.
     - Email address:
     - Password:
3. **For this section please make sure to use ChatGPT auto or GPT-4 model**


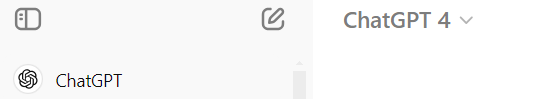

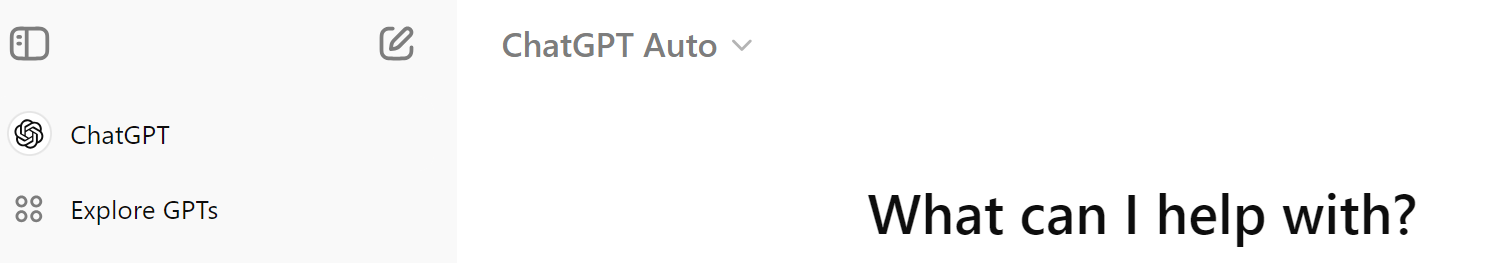


## Step 2: Start a New Chat

1. **Click on "New Chat"** on the left sidebar to begin a fresh conversation.

Tip: Starting a new chat for each assignment ensures previous interactions don't affect your current session.


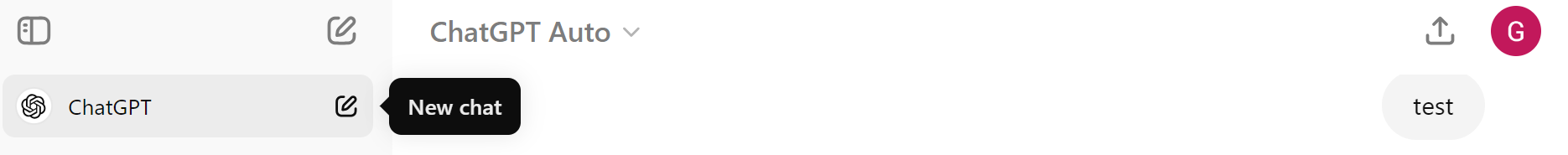


## Step 3: Prepare Your Assessment Instructions

**Locate the Assessment Instructions**

- Find the exact instructions provided for the assessment.
- Find the marking rubric used for the assessment.

## ****Step 4: Varying Quality Generation Using Chain Prompting****

In this section, we will apply different levels of chain prompting to generate varying quality answers from ChatGPT. Follow these steps to progressively refine the output based on the assessment instructions.

### **Quality 1: Basic Answer Generation**

1. **Make sure you are in the new chat window**
2. **Pasted the instruction**, type the following:

“Generate a report and in-text references according to the following specification.”

1. **Paste the copied assessment instructions** into the chat input box below the instruction.

**Tip: you can use “Shift+Enter” to go to next line inside of the chat box**


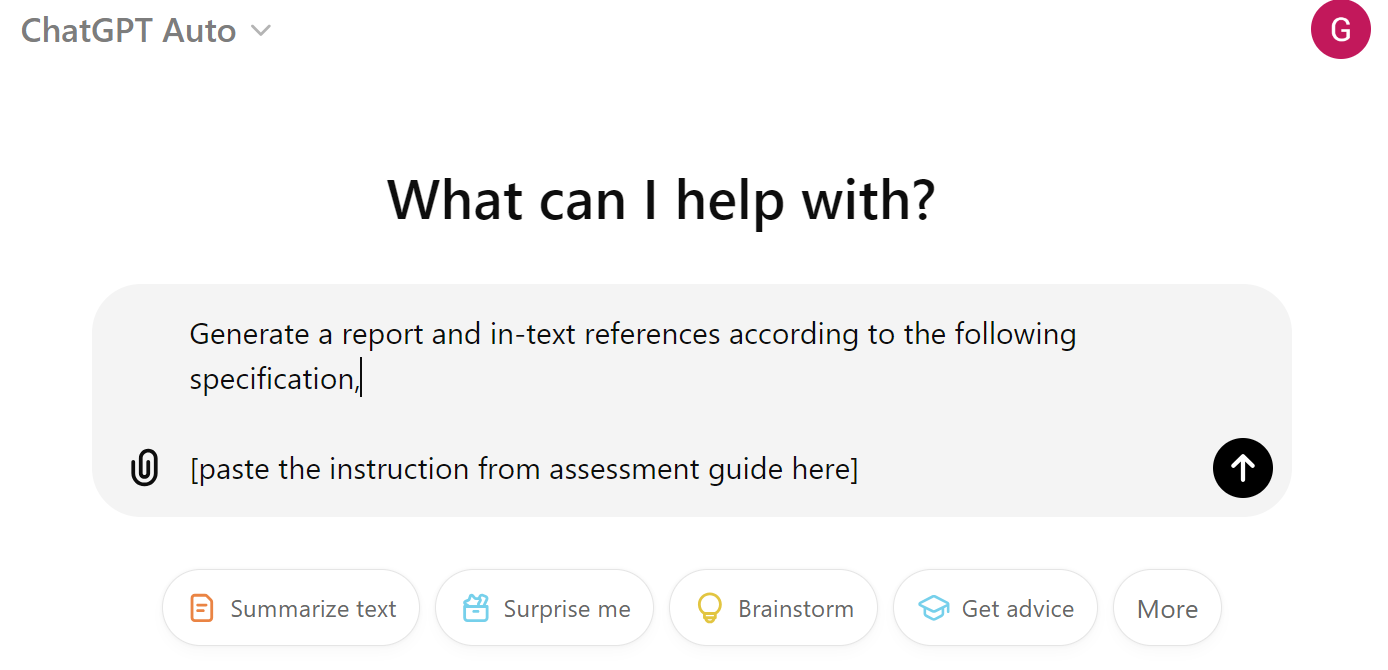


1. **Copy ChatGPT answer** into MS word document and save it as ”*subject code_01_date”*
2. **Rename the chat using the word document name**


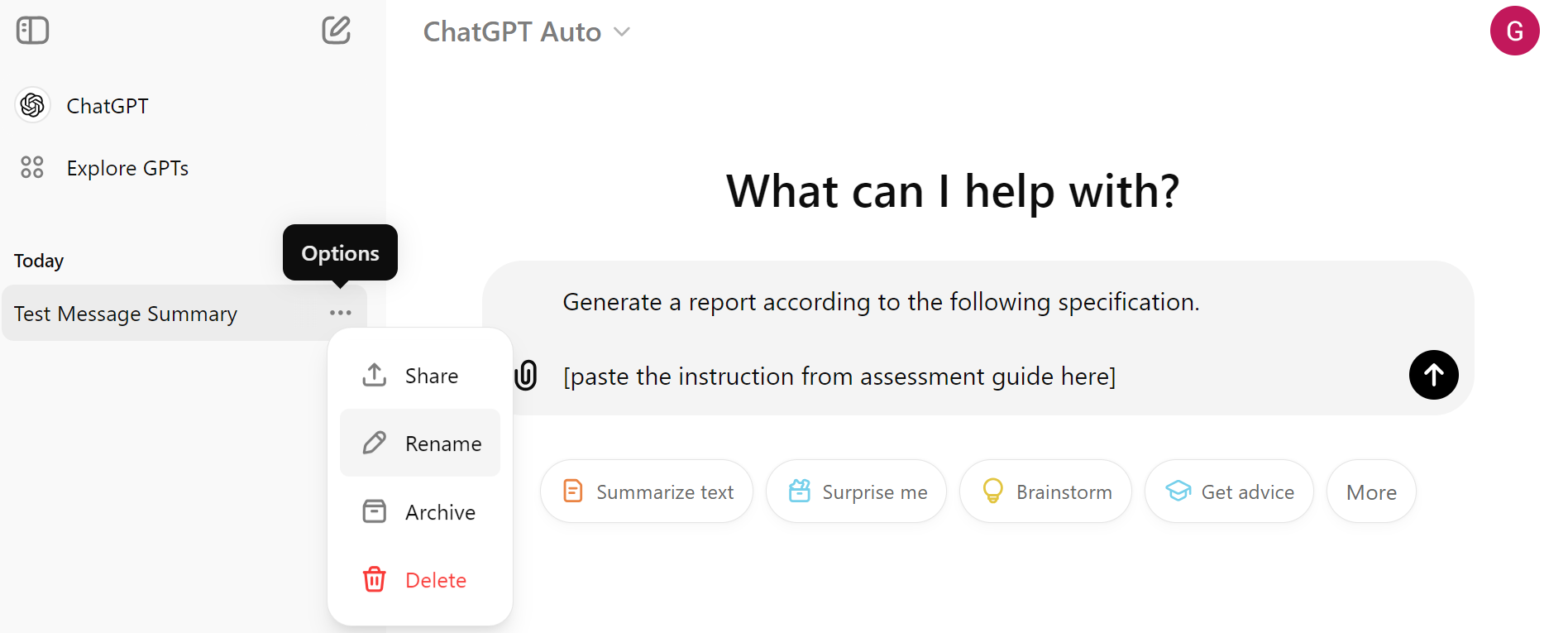


### Quality 2: Section-Based Refinement

1. **Make sure you are in the new chat window**
2. **Break down the instructions into sections**, such as Introduction, Literature Review, Methodology, etc.
3. Identify the specific sections required by the assessment instructions.
4. **Input the following prompt**:

“Generate a report and in-text references according to the following specification.”

“write the following section of the report“

1. **Paste the copied assessment instructions for the section** into the chat box below the instruction.


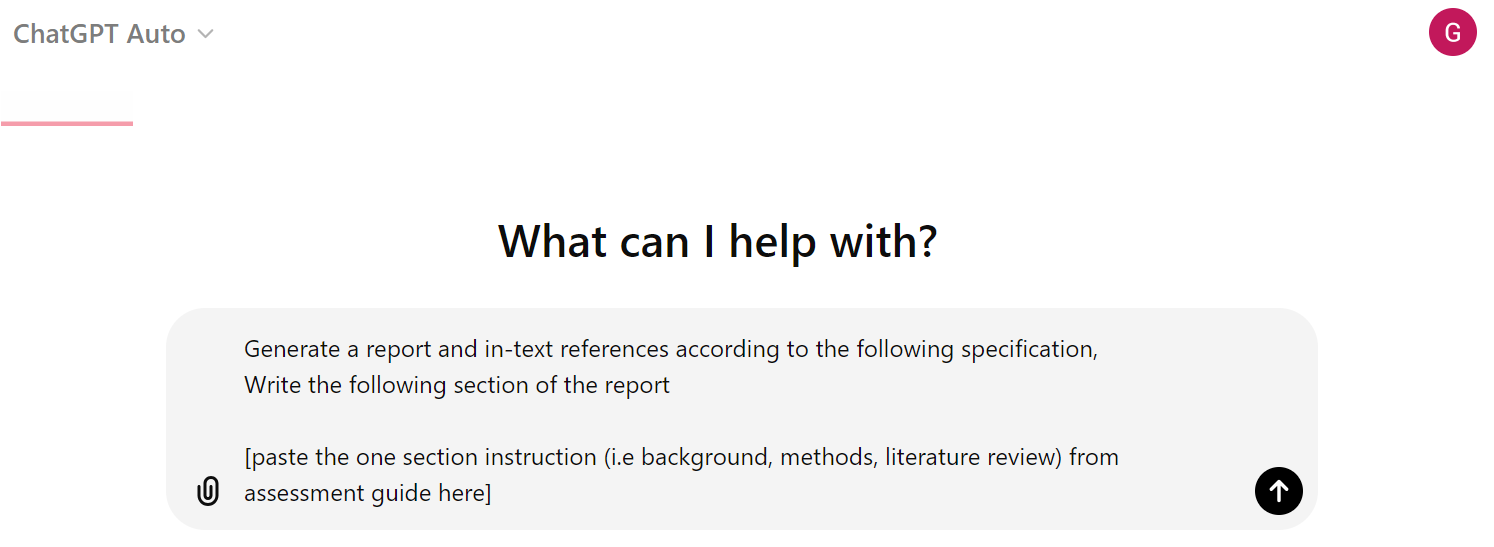


1. After completing all the sections required by the assessment instruction, **combine them in one document and save it** as ”*subject code_02_date”*
2. **Rename the chat using the word document name**

### Quality 3: Rubric-Based Refinement

1. **Make sure you are in the new chat window**
2. **Break down the instructions into sections**, such as Introduction, Literature Review, Methodology, etc.
3. Identify the specific sections required by the assessment instructions.
4. **Input the following prompt**:

“Generate a report and in-text references according to the following specification.”

“write the following section of the report“

1. **Paste the copied assessment instructions for the section** into the chat box below the instruction.
2. **Input the following prompt:**“Use the following criteria to enhance the report and ensure alignment with the grading rubric below.”
3. **Paste the rubric or grading criteria below the prompt above. See figure below for more clarity.**


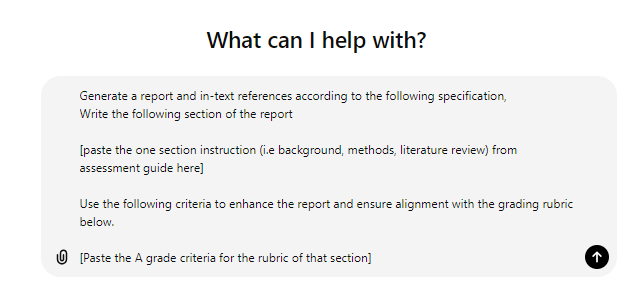


1. After completing all the sections required by the assessment instruction, **combine them in one document and save it** as ”*subject code_03_date”*
2. **Rename the chat using the word document name**

### Quality 4: Rubric-Based Refinement

1. **For this section please make sure to use GPT-o1 Preview**

**
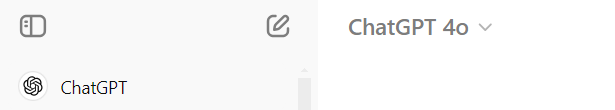
**

1. **Similar to quality 3, you will need to Break down the instructions into sections**, such as Introduction, Literature Review, Methodology, etc.
2. Identify the specific sections required by the assessment instructions.
3. **Input the following prompt**:

“Generate a report and in-text references according to the following specification.”

“write the following section of the report“

1. **Paste the copied assessment instructions for the section** into the chat box below the instruction.
2. **Input the following prompt:**“Use the following criteria to enhance the report and ensure alignment with the grading rubric below.”
3. **Paste the rubric or grading criteria below the prompt above.** See figure below for more clarity.


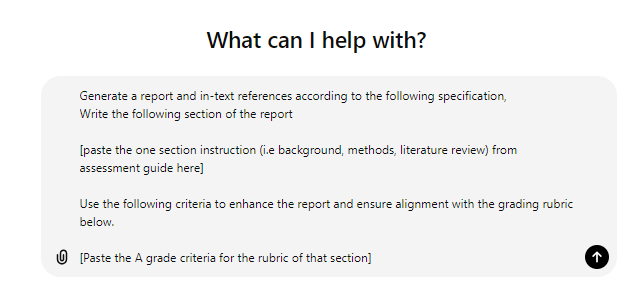


1. After completing all the sections required by the assessment instruction, **combine them in one document and save it** as ”*subject code_04_date”*
2. **Rename the chat** using the word document name
